# Supplementary material for: Development of clinical decision rules for traumatic intracranial injuries in patients with mild traumatic brain injury in a developing country
Source: PLoS One. 2020 Sep 18;15(9):e0239082. doi: 10.1371/journal.pone.0239082 (PMC7500687; doi:10.1371/journal.pone.0239082)
Supplement: S1 Table — (DOCX) [file pone.0239082.s001.docx]

**S1 Table.** Detailed results regarding road traffic injuries and other mechanism of injuries

S1

| **Mechanism of injury** | **Intracranial injuries on CT scan** | | | | **Total**  **(n=1164)** | |
| --- | --- | --- | --- | --- | --- | --- |
|  | **Present**  **(n=244)** | | **Absent**  **(n=920)** | |  |  |
|  | n | (%) | n | % | n | (%) |
| Motorcycle accident |  |  |  |  |  |  |
| Did not wear helmet | 148 | 60.7 | 433 | 47.1 | 581 | 49.9 |
| Wear helmet | 5 | 2.0 | 66 | 7.2 | 71 | 6.1 |
| Car accident |  |  |  | 0.0 |  |  |
| Not ejected from vehicle | 1 | 0.4 | 37 | 4.0 | 38 | 3.3 |
| Ejected from vehicle | 7 | 2.9 | 8 | 0.9 | 15 | 1.3 |
| Pedestrians hit by a car | 4 | 1.6 | 13 | 1.4 | 17 | 1.5 |
| Bicycle hit by a car | 0 | 0.0 | 1 | 0.1 | 1 | 0.1 |
| Falling from height |  |  |  | 0.0 |  |  |
| <1 meter | 27 | 11.1 | 170 | 18.5 | 197 | 16.9 |
| 1-3 meters | 10 | 4.1 | 14 | 1.5 | 24 | 2.1 |
| > 3 meters | 6 | 2.5 | 4 | 0.4 | 10 | 0.9 |
| unknown height | 5 | 2.0 | 54 | 5.9 | 59 | 5.1 |
| Physical assault | 21 | 8.6 | 85 | 9.2 | 106 | 9.1 |
| Head struck by an object | 4 | 1.6 | 14 | 1.5 | 18 | 1.5 |
| Fall from bicycle | 4 | 1.6 | 11 | 1.2 | 15 | 1.3 |
| Head collision while walking | 0 | 0.0 | 2 | 0.2 | 2 | 0.2 |
| Unknown mechanism | 2 | 0.8 | 8 | 0.9 | 10 | 0.9 |

Abbreviations: CT, computed tomography
