# Supplementary material for: Development of clinical decision rules for traumatic intracranial injuries in patients with mild traumatic brain injury in a developing country
Source: PLoS One. 2020 Sep 18;15(9):e0239082. doi: 10.1371/journal.pone.0239082 (PMC7500687; doi:10.1371/journal.pone.0239082)
Supplement: S2 Table — (DOCX) [file pone.0239082.s002.docx]

**S2 Table**. Detailed results of internal validation with the use of bootstrap resampling procedure

| **Variable** | **Obs** | **Mean** | **Standard deviation** | **Minimum** | **Maximum** |
| --- | --- | --- | --- | --- | --- |
| **For prediction of positive CT scans** | | | | | |
| Apparent AuROC | 500 | 0.850968 | 0.013631 | 0.806305 | 0.890054 |
| Apparent CITL | 500 | 0.000975 | 0.042948 | -0.15735 | 0.109892 |
| Apparent slope | 500 | 5.76796 | 0.2593 | 5.066488 | 6.486537 |
| Test AuROC | 500 | 0.845027 | 0.002091 | 0.83881 | 0.849425 |
| Test CITL | 500 | -0.00162 | 0.042849 | -0.11577 | 0.152206 |
| Test slope | 500 | 0.981076 | 0.032894 | 0.893679 | 1.085253 |
| Optimism AuROC | 500 | 0.005941 | 0.013521 | -0.042 | 0.0436 |
| Optimism CITL | 500 | 0.002598 | 0.085788 | -0.30955 | 0.22566 |
| Optimism slope | 500 | 0.018924 | 0.032894 | -0.08525 | 0.106322 |
| **For prediction of neurosurgical intervention requirements** | | | | | |
| Apparent AuROC | 500 | 0.874451 | 0.019177 | 0.817368 | 0.928808 |
| Apparent CITL | 500 | -2.2496 | 0.165259 | -2.74325 | -1.77763 |
| Apparent slope | 500 | 4.996052 | 0.476889 | 3.596919 | 6.590736 |
| Test AuROC | 500 | 0.87144 | 0.003507 | 0.85583 | 0.879951 |
| Test CITL | 500 | -2.24407 | 0.062564 | -2.39543 | -2.04466 |
| Test slope | 500 | 0.884638 | 0.038847 | 0.772182 | 0.998251 |
| Optimism AuROC | 500 | 0.003011 | 0.019297 | -0.05233 | 0.053454 |
| Optimism CITL | 500 | -0.00553 | 0.171746 | -0.50864 | 0.603689 |
| Optimism slope | 500 | 0.115362 | 0.038847 | 0.00175 | 0.227818 |

Abbreviations: Obs, observations (number of replicates); AuROC, area under receiver operating characteristic curve; CITL, calibration in the large
