# Supplementary material for: Development of clinical decision rules for traumatic intracranial injuries in patients with mild traumatic brain injury in a developing country
Source: PLoS One. 2020 Sep 18;15(9):e0239082. doi: 10.1371/journal.pone.0239082 (PMC7500687; doi:10.1371/journal.pone.0239082)
Supplement: S3 Table — (DOCX) [file pone.0239082.s003.docx]

**S3 Table.** Sensitivity analysis of the model-based prediction score between standard logistic regression and multi-level logistic regression analysis

| **Predictors** | **Standard logistic regression** | | | **Multi-level logistic regression** | | |
| --- | --- | --- | --- | --- | --- | --- |
|  | **OR** | **95%CI** | **p-value** | **OR** | **95%CI** | **p-value** |
| Presence of neurological deficits | 18.3 | 2.6,126.3 | 0.003 | 37.3 | 4.1,337.3 | 0.001 |
| Diffuse headache | 5.8 | 4.0,8.2 | <0.001 | 3.2 | 2.1,4.7 | <0.001 |
| Signs of skull base fracture | 4.8 | 2.8,8.5 | <0.001 | 2.8 | 1.6,5.0 | <0.001 |
| GCS <15 (at 2 hours) | 4.4 | 2.5,7.8 | <0.001 | 7.0 | 3.6,13.8 | <0.001 |
| Dangerous mechanisms | 4.1 | 1.7,10.0 | <0.001 | 3.3 | 1.3,8.2 | 0.010 |
| Stepping of the skull | 3.8 | 1.8,7.9 | <0.001 | 3.0 | 1.4,6.4 | 0.004 |
| Wound at the scalp | 2.7 | 1.9,4.0 | <0.001 | 2.1 | 1.4,3.1 | <0.001 |
| Vomiting >2 episodes | 2.0 | 1.2,3.3 | 0.013 | 2.8 | 1.6,5.1 | 0.001 |
| Current anticoagulant use | 1.6 | 0.5,5.5 | 0.451 | 2.2 | 0.5,8.8 | 0.272 |
| Posttraumatic amnesia | 1.3 | 0.9,1.9 | 0.237 | 1.5 | 1.0,2.2 | 0.057 |
| Age ≥60 years | 1.3 | 0.8,2.0 | 0.357 | 1.8 | 1.1,3.1 | 0.026 |
| Posttraumatic seizure | 1.1 | 0.5,2.2 | 0.902 | 1.9 | 0.9,4.3 | 0.106 |
| Loss of consciousness | 1.1 | 0.7,1.5 | 0.912 | 1.0 | 0.7,1.6 | 0.902 |
| Model Intercept | 0.03 | 0.02,0.05 | <0.001 | 0.02 | 0.004,0.16 | <0.001 |
| AuROC before score transformation | 0.85 | 0.82,0.87 |  | 0.89 | 0.87,0.92 |  |
| AuROC after score transformation | 0.85 | 0.82,0.87 |  | 0.84 | 0.81,0.87 |  |

Abbreviations: OR, odds ratio; CI, confidence interval; ß, beta-coefficient; GCS, Glasgow Coma Scale; SE, standard error; Min, minimum; Max, maximum; CT, computed tomography.

S3
